# Ensemble learning predicts multiple sclerosis disease course in the SUMMIT study

**DOI:** 10.1038/s41746-020-00338-8

**Published:** 2020-10-16

**Authors:** Yijun Zhao, Tong Wang, Riley Bove, Bruce Cree, Roland Henry, Hrishikesh Lokhande, Mariann Polgar-Turcsanyi, Mark Anderson, Rohit Bakshi, Howard L. Weiner, Tanuja Chitnis, SUMMIT Investigators

**Affiliations:** 1 Department of Computer and Information Science, Fordham University, New York, NY USA; 2 University of California, San Francisco, CA USA; 3 SUMMIT Consortium, Boston, MA USA; 4 SUMMIT Consortium, San Francisco, CA USA; 5 Brigham Multiple Sclerosis Center, Ann Romney Center, Brigham and Women’s Hospital, Harvard Medical School, Boston, MA USA

**Keywords:** Multiple sclerosis, Multiple sclerosis

## Abstract

The rate of disability accumulation varies across multiple sclerosis (MS) patients. Machine learning techniques may offer more powerful means to predict disease course in MS patients. In our study, 724 patients from the Comprehensive Longitudinal Investigation in MS at Brigham and Women’s Hospital (CLIMB study) and 400 patients from the EPIC dataset, University of California, San Francisco, were included in the analysis. The primary outcome was an increase in *Expanded Disability Status Scale* (*EDSS*) ≥ 1.5 (worsening) or not (non-worsening) at up to 5 years after the baseline visit. Classification models were built using the CLIMB dataset with patients’ clinical and MRI longitudinal observations in first 2 years, and further validated using the EPIC dataset. We compared the performance of three popular machine learning algorithms (*SVM, Logistic Regression,* and *Random Forest*) and three ensemble learning approaches (*XGBoost, LightGBM*, and a Meta-learner *L*). A “threshold” was established to trade-off the performance between the two classes. Predictive features were identified and compared among different models. Machine learning models achieved 0.79 and 0.83 AUC scores for the CLIMB and EPIC datasets, respectively, shortly after disease onset. Ensemble learning methods were more effective and robust compared to standalone algorithms. Two ensemble models, XGBoost and LightGBM were superior to the other four models evaluated in our study. Of variables evaluated, EDSS, *Pyramidal Function*, and *Ambulatory Index* were the top common predictors in forecasting the MS disease course. Machine learning techniques, in particular ensemble methods offer increased accuracy for the prediction of MS disease course.

## Introduction

The majority of currently approved multiple sclerosis (MS) therapies primarily target relapses, and have limited effects on halting the overall disability progression. Although a number of clinical and demographic features have been associated with long-term disease course in MS^1–7^, there is increasing evidence that early and more aggressive treatment targeting relapses may delay or prevent the long-term accumulation of disability^8,9^, but this effect must be balanced with the potential increase in side effects associated with more potent therapies. The identification of patients who are more likely to accrue disability would allow clinicians to institute more rigorous monitoring procedures and potentially initiate more potent therapies early in the course of the disease.

In our research, we apply machine learning techniques to predict the disability level of MS patients at the five-year time point using the first two years of clinical and neuroimaging longitudinal data. The level of MS disability is measured by the *Expanded Disability Status Scale* (*EDSS*) score^10^ using a 0–10 scale, in which 0 is normal and 6 corresponds to walking with a cane. Our goal is to predict which patients will accumulate disability (“worsening”), and which are likely to remain without disability accumulation (“non-worsening”) in their disease course. We define “worsening” as an increase of 1.5 or more from the baseline EDSS to the 5-year EDSS, and “non-worsening” as all other cases. The threshold is selected based on the fact that an EDSS increase of 1.0 or 1.5 is clinically significant and generally sustained, and is used as a primary or secondary endpoint in clinical trials of MS therapies.

In this paper, we present our findings by applying ensemble techniques to integrate information from multiple machine learning classifiers. Ensemble learning has been proven to produce better and more robust predictive performance compared to any single model. In our experiment, we created a heterogeneous Meta-learner *L* from three established machine learning algorithms as our base classifiers: *Support Vector Machines* (*SVM*), *Logistic Regression*, and *Random Forest*. We further investigated the efficacy of two more homogeneous ensemble learners, *XGBoost* and *LightGBM*, which have gained much attention in recent years due to their superior performance^11,12^.

An additional motivation for our research is to study risk factors affecting MS patients’ disease progressions. To this end, we ranked the top predictors in our models and identified the most predictive factors. Detailed findings and discussions are presented in the “Results” section.

## Results

### Model performance

All experiments were conducted by running a nested cross-validation. Specifically, the outer loop splits the data into ten stratified nonoverlapping folds. Each of the ten folds will subsequently be held out as the test data, while the remaining folds form the training data. For each training set, we apply a nested 10-fold cross-validation to select the hyper-parameters via a grid search based on the highest AUC (area under the ROC curve) score. We report the average model performance of the outer ten test folds. In addition to overall predictive accuracy, sensitivity and specificity were used to measure the performance in the positive and negative classes, respectively.

Table 1 presents our experimental results on the Comprehensive Longitudinal Investigation in MS at Brigham and Women’s Hospital (CLIMB) dataset using 6-month observation windows. Since we are more interested in predicting the “worsening” class, we applied different thresholds in the ROC curve to increase a model’s sensitivity at the cost of lowering the specificity. For each threshold displayed in column 1 of Table 1, we present a performance comparison of the six models described in the “Methods” section, using sensitivity, specificity, and overall accuracies. Consequently, we can observe the trade-offs between an increase in sensitivity and a decrease in specificity for each model, as we shift the threshold. A healthcare institution can select a desired threshold depending on its level of tolerance on the insufficient performance of the “non-worsening” class (i.e., a low specificity). From Table 1, we observe that:

- The highest accuracy on the “worsening” class that is of practical value is about 80%. This is because further improvement would lead to a <50% performance of the “non-worsening” class. Given 80% as the benchmark on the “worsening” class, XGBoost and LightGBM are the best models, with each achieving close to 70% on the other class at thresholds 0.35 and 0.3, respectively. Meta-learner *L* is the next runner-up with 65% accuracy on the “non-worsening” class.
- It is worth noting that some algorithms are more sensitive to the shift of threshold values. For example, Random Forest degenerated quickly as the threshold value moved <0.4. On the other hand, XGBoost and LightGBM maintained a steady trade-off between the two classes, as we varied the thresholds. We conclude that they are the desirable models for our task due to their superior performance and robustness.

**Table 1:** ML models applied to the CLIMB dataset with varying thresholds.

Threshold	Model	Sensitivity	Specificity	Overall
0.5	SVM	0.60	0.70	0.68
	Logistic Regression	0.70	0.71	0.71
	Random Forest	0.72	0.73	0.73
	XGBoost	0.50	0.87	0.79
	LightGBM	0.51	0.86	0.78
	Meta-L^a^	0.61	0.84	0.79
0.45	SVM	0.75	0.64	0.67
	Logistic Regression	0.76	0.62	0.65
	Random Forest	0.83	0.51	0.58
	XGBoost	0.58	0.82	0.77
	LightGBM	0.52	0.85	0.77
	Meta-L^a^	0.71	0.74	0.73
0.4	SVM	0.81	0.51	0.58
	Logistic Regression	0.81	0.57	0.62
	Random Forest	0.91	0.34	0.47
	XGBoost	0.68	0.76	0.74
	LightGBM	0.58	0.82	0.77
	Meta-L^a^	**0.78**	**0.65**	**0.68**
0.35	SVM	0.92	0.34	0.47
	Logistic Regression	0.86	0.49	0.57
	Random Forest	0.98	0.11	0.31
	XGBoost	**0.79**	**0.69**	**0.71**
	LightGBM	0.70	0.76	0.75
	Meta-L^a^	0.86	0.50	0.58
0.3	SVM	0.96	0.21	0.38
	Logistic Regression	0.91	0.41	0.52
	Random Forest	0.99	0.06	0.27
	XGBoost	**0.81**	**0.64**	**0.68**
	LightGBM	**0.78**	**0.68**	**0.70**
	Meta-L^a^	0.93	0.35	0.48

Bold numbers indicate models of high practical value.

^a^Ensemble of SVM, Logistic Regression, and Random Forest.

Table 2 presents the validation results of our models using the EPIC dataset. To facilitate a validation from a dataset with different variables and data frequency, we rebuilt our models using the CLIMB dataset, but with only the overlapping attributes of the two datasets and with annual observations. The resulting models were applied directly to the EPIC dataset to evaluate the efficacy of our models. We first observe that, similar to Table 1, ensemble methods continue to be the top performers for the CLIMB dataset. However, the best attainable performance has decreased to ~75 and 61% for the “worsening” and “non-worsening” classes, respectively. The reduced predicability is expected because the results in Table 2 were obtained using fewer variables and less data frequency than those in Table 1. We further observe that the desired models for the CLIMB data align with the ones for the EPIC dataset.

**Table 2:** Model validation using overlapping attributes and annual observations.

Threshold	Model	Sensitivity		Specificity		Overall	
		CLIMB	EPIC	CLIMB	EPIC	CLIMB	EPIC
0.5	SVM	0.63	0.81	0.75	0.70	0.72	0.74
	Logistic Regression	0.64	0.76	0.78	0.72	0.75	0.73
	Random Forest	0.62	0.83	0.77	0.65	0.74	0.71
	XGBoost	0.58	0.75	0.75	0.71	0.71	0.72
	LightGBM	0.56	0.62	0.75	0.83	0.71	0.76
	Meta-L^a^	0.61	0.78	0.79	0.76	0.75	0.77
0.45	SVM	**0.76**	0.90	**0.61**	0.45	0.64	0.60
	Logistic Regression	0.69	0.83	0.69	0.65	0.69	0.71
	Random Forest	0.73	0.90	0.63	0.53	0.65	0.65
	XGBoost	0.68	0.79	0.70	0.66	0.70	0.70
	LightGBM	0.69	0.69	0.68	0.77	0.68	0.74
	Meta-L^a^	0.70	0.85	0.68	0.70	0.68	0.75
0.4	SVM	0.84	0.93	0.47	0.42	0.55	0.59
	Logistic Regression	**0.78**	**0.88**	**0.60**	**0.59**	**0.64**	**0.68**
	Random Forest	0.85	0.92	0.54	0.39	0.61	0.56
	XGBoost	**0.75**	**0.85**	**0.62**	**0.60**	**0.65**	**0.68**
	LightGBM	**0.75**	**0.73**	**0.61**	**0.73**	**0.64**	**0.73**
	Meta-L^a^	**0.81**	**0.90**	**0.58**	**0.58**	**0.63**	**0.68**
0.35	SVM	0.92	0.96	0.37	0.32	0.50	0.53
	Logistic Regression	0.86	0.92	0.51	0.51	0.59	0.64
	Random Forest	0.89	0.96	0.45	0.31	0.55	0.52
	XGBoost	0.85	**0.87**	0.54	**0.60**	0.61	0.69
	LightGBM	0.85	**0.80**	0.52	**0.70**	0.60	0.73
	Meta-L^a^	0.88	0.93	0.49	0.52	0.58	0.65
0.3	SVM	0.93	0.98	0.25	0.23	0.40	0.47
	Logistic Regression	0.90	0.93	0.41	0.48	0.52	0.63
	Random Forest	0.95	0.95	0.30	0.24	0.45	0.47
	XGBoost	0.90	0.90	0.45	0.56	0.55	0.67
	LightGBM	0.92	0.86	0.42	0.62	0.53	0.70
	Meta-L^a^	0.93	0.96	0.38	0.37	0.51	0.56
Regression coef. (*p* value) *R*-square (correlation)		1.08 (6.9E−08) 0.65 (0.81)		0.77 (8.6E−09) 0.70 (0.84)		0.88 (1.8E−08) 0.68 (0.83)	

Bold numbers indicate models of high practical value.

^a^Ensemble of SVM, Logistic Regression, and Random Forest.

It is worth noting that SVM and Logistic Regression achieved similar performance, as the ensemble methods for the CLIMB dataset in Table 2. However, SVM didn’t sustain its effectiveness for the EPIC dataset and Logistic Regression was not a favored model in Table 1. Thus, we recommend ensemble models for our classification task due to their robust performance across varied datasets and experiments.

In addition to validating the optimal models across the two datasets, we further evaluated the overall similarity of model performance for all thresholds. To this end, we performed an independent regression analysis for each evaluation metric (i.e., sensitivity, specificity, and overall), using the corresponding accuracies of the two datasets. We present the regression statistics in the last two rows of Table 2. The high *R*-square (correlation) values indicate that our models’ performances in the two datasets are highly similar. This is further evidenced by the regression coefficients where a value closer to one indicates a better match. In addition, the nearly zero *p* values imply the statistical significance of the coefficients.

Lastly, we present the AUC scores for our experiments in Table 3. The “CLIMB-all” and “CLIMB-part” columns denote the models trained using all and partial CLIMB features, respectively. In each column, all machine learning models differ marginally in terms of the AUC metric. However, AUC measures a model’s effectiveness over all thresholds, including the ones without practical values. The above closer analysis of Tables 1 and 2 revealed that the ensemble algorithms produced the most useful results for the two datasets in both experiments. We further observe that the models achieved higher AUC scores on the validation (EPIC) dataset, which confirms the generalizability of our models.

**Table 3:** AUC scores of six models across the two dataset.

Model	CLIMB_all^a^	CLIMB_part^b^	EPIC
SVM	0.75	0.76	0.81
Logistic Regression	0.78	0.77	0.81
Random Forest	0.77	0.77	0.82
XGBoost	0.78	0.76	0.82
LightGBM	0.78	0.76	0.82
Meta-*L*	0.79	0.78	0.83

^a^Models trained using complete CLIMB data.

^b^Models trained using overlapping features of CLIMB and EPIC datasets, and annual observations.

### Risk factor analysis

We next examined the major factors that are predictive of MS progression. Five linear and tree-based algorithms, SVM, Random Forest, Logistic Regression, XGBoost, and LightGBM were selected for the study. These models were chosen because their feature importance was well defined. For linear models, the importance of a feature is proportional to the magnitude of its coefficients. Specifically, all linear models can be expressed as a linear combination of the dependent variables^13^, i.e.,

1 $$y = \omega _0 + \omega _1x_1 + \omega _2x_2 + \ldots + \omega _Dx_D,$$

where *y* is the target, (*x*_1_, *x*_2_, …, *x*_*D*_) are the dependent variables, and (*ω*_0_, *ω*_1_, …, *ω*_*D*_) are the model parameters. With a preprocessed dataset, where each feature *x*_*i*_ is normalized across all samples, the magnitude of *ω*_*i*_ indicates the contribution of *x*_*i*_ in making the prediction.

For *Decision Tree* (DT)-based models, the ranking follows the order of attributes that the algorithm chooses to split the branches. The algorithm implicitly performs feature selection by selecting an available node that produces the most homogeneous (i.e., purest) subbranches using criteria, such as *Information Gain or Gini Index*^14^. For a tree-based ensemble algorithm, attributes are ranked according to their average rank scores across all trees.

Table 4 presents the top ten risk factors identified by each of the five models, using the CLIMB dataset.

- Examining the top five risk factors associated with each model, we identified two consistent principal predictors (highlighted in bold) across all models. The first one, as expected, is either the EDSS score or its progression. It is worth noting that our task is to predict a patient’s EDSS score at 5-year mark, using the first 2-year observations. Thus, these EDSS-related predictors are lagged observations at the onset of the disease. The second principal predictor is a patient’s pyramidal function measure. In addition, a patient’s MS disease category or its progression is another important variable that appeared in four out of the five models. Specifically, SVM and Logistic Regression are dependent on ∆*DISEASE_CATEGORY*, while XGBoost and LightGBM rely on the value of DISEASE_CATEGORY itself.

- Expanding our investigation to the top ten risk factors associated with each model, we could identify two more common risk factors across all models, namely *DISEASE_ACTIVITY* and *AMBULATORY_INDEX* (or its related change ∆AMBULATORY_INDEX).
- In addition to the seven common risk factors revealed by all models, the measure of a patient’s bowel and bladder function is the next important risk factor to watch out for because it appeared in three out of the five models.
- Furthermore, SVM, Logistic Regression, and Random Forest rely on a patient’s total number of Gad+ lesions and TDS+ calculated brain parenchymal fraction in making their predictions, whereas XGBoost and LightGBM utilize a patient’s genetic information, i.e., *FAMILY_MS*, in their decisions.

**Table 4:** Top ten predictive features identified by five models using the CLIMB dataset.

Rank	SVM	Logistic Regression	Random Forest
1	**∆EDSS**	**∆EDSS**	**∆EDSS**
2	**PYRAMIDAL_FUNCTION**	**PYRAMIDAL_FUNCTION**	**EDSS**
3	∆LESION_VOLUME	∆AMBULATORY_INDEX	**PYRAMIDAL_FUNCTION**
4	∆DISEASE_CATEGORY	MRI_STATUS	AMBULATORY_INDEX
5	∆AMBULATORY_INDEX	∆DISEASE_CATEGORY	DISEASE_ACTIVITY
6	AMBULATORY_INDEX	BOWEL_BLADDER_FUNCTION	DISEASE_STEP
7	BOWEL_BLADDER_FUNCTION	DISEASE_ACTIVITY	∆AMBULATORY_INDEX
8	∆TOTAL_GD	∆TOTAL_GD	∆SENSORY_FUNCTION
9	DISEASE_ACTIVITY	AMBULATORY_INDEX	DISEASE_CATEGORY
10	∆WALKING_ABILITY	DISEASE_COURSE_SUBTYPE	∆BPF

Rank	XGBoost	LightGBM	
1	**∆EDSS**	**EDSS**	
2	**EDSS**	**∆EDSS**	
3	DISEASE_CATEGORY	DISEASE_CATEGORY	
4	MRI_STATUS	MRI_STATUS	
5	**PYRAMIDAL_FUNCTION**	**PYRAMIDAL_FUNCTION**	
6	ATTACKPREV2Y	AMBULATORY_INDEX	
7	FAMILY_MS	ATTACKPREV2Y	
8	AMBULATORY_INDEX	FAMILY_MS	
9	DISEASE_ACTIVITY	BOWEL_BLADDER_FUNCTION	
10	VISIT_AGE	DISEASE_ACTIVITY	

∆: change in the indicated variable.

AMBULATORY_INDEX: ordinal scale of gait capacity.

ATTACKPREV2Y: number of clinical relapses (attacks) in the previous 2 years.

BOWEL_BLADDER_FUNCTION: measure of bowel and bladder function from 0 (normal) to 6 (loss of bowel and bladder function).

DISEASE_ACTIVITY: physician reported metric of current inflammatory or progressive disease status.

DISEASE_CATEGORY: code indicating disease categories, such as primary progressive, secondary progressive, etc.

DISEASE_STEP: scale of disability.

EDSS: overall neurologic disability score.

FAMILY_MS: code indicating family history of MS, including mother, father, sibling, cousin, etc.

LESION_VOLUME: brain T2 lesion volume measured.

MRI_STATUS: presence of new MRI lesions.

PYRAMIDAL_FUNCTION: measure of pyramidal function from 0 (normal) to 6.

SENSORY_FUNCTION: measure of sensory disability ranging from 0 (normal) to 6 (sensation lost below the head).

BPF: brain parenchymal fraction.

TOTAL_GD: total number of Gad+ lesions.

VISIT_AGE: age of the subject.

Table 5 presents the top ten risk factors identified by each of the five models using the EPIC dataset.

- Examining the top five risk factors identified by each model, we observe that the EPIC dataset displayed same top two consistent principal predictors (i.e., EDSS and *PYRAMIDAL_FUNCTION*) like the CLIMB dataset across all models.
- Expanding to the top ten risk factors, we observe that the volume of the cerebrospinal fluid and the brain gray matter volume are two major predictors for the EPIC dataset. These two variables were not included in the CLIMB dataset. We recommend including them in the future CLIMB data collection effort.
- Other major predictors identified for the EPIC dataset are *AGE*, *MENTAL_FUNCTION*, and *CEREBELLAR_FUNCTION*. Although these variables are among the risk factors presented in Table 4, their rankings are lower than other factors, including DISEASE_CATEGORY*,* DISEASE_ACTIVITY, and AMBULATORY_INDEX. Noting that the latter ones were not present in the EPIC dataset, we recommend including them in the future EPIC data collection effort.

**Table 5:** Top ten predictive features identified by five models using the EPIC dataset.

Rank	SVM	Logistic Regression	Random Forest
1	∆EDSS	∆EDSS	∆EDSS
2	BRAIN_GREY_VOLUME	∆PYRAMIDAL_FUNCTION	EDSS
3	CEREBELLAR_FUNCTION	VISIT_AGE	∆PYRAMIDAL_FUNCTION
4	∆PYRAMIDAL_FUNCTION	VENTRICULAR_CSF_VOLUME	PYRAMIDAL_FUNCTION
5	ATTACKPREV2Y	CEREBELLAR_FUNCTION	BRAIN_WHITE_VOLUME
6	PYRAMIDAL_FUNCTION	ATTACKPREV2Y	SENSORY_FUNCTION
7	VENTRICULAR_CSF_VOLUME	∆MENTAL_FUNCTION	VENTRICULAR_CSF_VOLUME
8	VISIT_AGE	MENTAL_FUNCTION	∆BOWEL_BLADDER_FUNCTION
9	∆BRAIN_GREY_VOLUME	PYRAMIDAL_FUNCTION	TIMED_WALK_TRIAL
10	MENTAL_FUNCTION	BRAIN_GREY_VOLUME	BRAIN_GREY_VOLUME

Rank	XGBoost	LightGBM	
1	∆EDSS	∆EDSS	
2	EDSS	EDSS	
3	PYRAMIDAL_FUNCTION	PYRAMIDAL_FUNCTION	
4	∆PYRAMIDAL_FUNCTION	∆PYRAMIDAL_FUNCTION	
5	VISIT_AGE	VISIT_AGE	
6	ATTACKPREV2Y	ATTACKPREV2Y	
7	MENTAL_FUNCTION	CEREBELLAR_FUNCTION	
8	∆BOWEL_BLADDER_FUNCTION	∆BOWEL_BLADDER_FUNCTION	
9	CEREBELLAR_FUNCTION	VENTRICULAR_CSF_VOLUME	
10	VENTRICULAR_CSF_VOLUME	BRAIN_GREY_VOLUME	

∆: change in the indicated variable.

ATTACKPREV2Y: number of clinical relapses (attacks) in the previous 2 years.

BOWEL_BLADDER_FUNCTION: measure of bowel and bladder function from 0 (normal) to 6 (loss of bowel and bladder function).

BRAIN_GREY_VOLUME: total brain gray matter volume.

BRAIN_WHITE_VOLUME: total brain white matter volume.

CEREBELLAR_FUNCTION: measure of cerebella function from 0 (normal) to 5 (severe ataxia)

EDSS: overall neurologic disability score.

MENTAL_FUNCTION: measure of mental function from 0 (normal) to 5 (dementia).

PYRAMIDAL_FUNCTION: measure of pyramidal function from 0 (normal) to 6 (tetraplegia).

SENSORY_FUNCTION: measure of sensory function from 0 (normal) to 6 (loss of sensation below head).

TIMED_WALK_TRIAL: average time (in seconds) for two trials of the 25-foot walk.

VENTRICULAR_CSF_VOLUME: volume of the cerebrospinal fluid in the ventricles. In the EPIC study, this is usually reported in cm^3^.

VISIT_AGE: age of the subject.

## Discussion

In this study, we applied machine learning techniques to predict disability accumulation levels of MS patients at the 5-year mark based on 2-year clinical observations. We built and validated our models using two real-world datasets: 724 patients enrolled in the CLIMB study at Brigham and Women’s Hospital, and 400 patients from the EPIC dataset from the University of California, San Francisco. We employed three baseline machine learning models and three ensemble learners in our study. We further addressed the data imbalance issue by increasing the weights for the minority class. Our experimental results demonstrate that XGBoost and LightGBM offer comparable predictive power for our task, and their performances are more robust than the other four models across the two datasets.

In addition, we examined the top risk factors identified by our linear and tree-based models for both CLIMB and EPIC datasets. Several common as well as independent variables were identified from the two datasets, and future studies should consider evaluating these further. We conclude that a patient’s change in EDSS scores over the baseline value, pyramidal function measure, MS disease category, disease activity, ambulatory index, volume of the cerebrospinal fluid, and the brain gray matter volume are the top predictive indicators to forecast a patient’s disability level in 5 years.

For future work, we plan to explore time series models, such as recurrent neural networks to better capture the temporal dependencies in the longitudial data. We also plan to incorporate genetic information and additional biomarkers from patients’ medical records.

## Methods

### Datasets

In this section, we describe the datasets, experimental design, and machine learning methods we employed to conduct our study.

We included data from two prospectively followed cohorts that together form part of the SUMMIT Consortium^15^. Our first dataset consists of 724 patients enrolled in the CLIMB^16^. CLIMB patients undergo a complete neurological examination every six months, including measurement of EDSS. MRI procedures are performed on these patients on an annual basis. The dataset consists of 44 longitudinal and 24 demographic features. Of these, we excluded variables with excessive missing values as well as medication-related attributes. To reflect the change of a patient’s disease progression, we added a lagged variable for each clinical attribute, capturing the difference between the current and previous time points. Categorical features were further preprocessed using one-hot encoding, a technique in which an integer-encoded categorical variable is converted to a set of binary variables, each of which indicates a unique value of the category. One-hot encoding eliminates the artificial ordering introduced by the integer values that a machine learning algorithm could exploit erroneously. We applied one-hot encoding only to categorical variables, but not to ordinal variables whose rankings could provide useful information to the model. After preprocessing the data, we have a total of 198 features over a 2-year observation window.

Our second dataset consists of 400 patients from the EPIC dataset from the University of California, San Francisco. Unlike the CLIMB dataset, patients in this dataset were monitored annually using 35 periodical features and 10 demographic variables. After augmenting the selected clinical attributes with additional lagged variables and preprocessing the categorical features, we have a total of 105 features over a 2-year observation window.

### Imbalanced training data

Although we are more interested in predicting the “worsening” patients, they form the minority class in our training data. Indeed, we have 165 “worsening” cases out of a total of 724 training samples in the CLIMB dataset. We employed the cost-sensitive learning^17^ technique to address the class imbalance issue during our model training. In this approach, a higher cost (i.e., weight) is assigned to all minority instances to facilitate a larger penalty when any of them are misclassified. For each algorithm, the best weight was selected as a hyper-parameter using a nested tenfold cross-validation during the model training. Specifically, we conducted a grid search on a list of weights centered around the ratio of the majority to minority samples, and selected the one with the highest average AUC score on the ten test folds.

While treating the imbalanced training data prevents degenerated models in which the predictions are biased towards the majority class, accurate forecast for the “worsening” class is inherently more challenging than that for the “non-worsening” class at the onset of the disease. To address this issue, we further establish a probability “threshold” to classify an instance belonging to the “worsening” class. Consequently, a lower threshold leads to higher accuracy in the “worsening” class at the cost of lower accuracy in the “non-worsening” class. Practitioners can select the model at different thresholds depending on their preferred tolerance on the false positive rate.

### Study design

Although both of our datasets are collected for similar research purposes, the CLIMB data constitute patient records with semiannual clinical visits, while the EPIC data are with annual follow-ups. In addition, the two datasets exhibit <50% common demographic and clinical features. The CLIMB study subjects provide informed consent, and this study is approved by the Partners Human Research Committee. EPIC subjects provide informed consent and this study is approved by the University of California Human Research Protection Program.

Table 6 summarizes the differences between the two datasets and presents their overlapping variables. These discrepancies prevent us from conducting a straightforward model validation, using one dataset for the other. To address this issue and, nonetheless, not limiting our model evaluation only to the overlapping variables, we design our study in three steps as follows.

**Table 6:** Comparison of the CLIMB and EPIC datasets.

Category	CLIMB	EPIC	Common
# of subjects	724	400	n/a
# of “worsening” subjects	165	130	n/a
# of demographic features	24	10	5
# longitudinal features	44	35	14
Clinical visit frequency	6 months	12 months	12 months
Common features			
Demographic features	Age	Gender	Smoking history
	Ethnicity	Race	
Longitudinal features	Attack previous 6 m	Disease category	Sensory function
	Attack previous 2Y	EDSS	Total GD
	Bowel–bladder function	Lesion volume	Visual function
	Brainstem function	Mental function	Walk 25 ft time
	Cerebellar function	Pyramidal function	

Step 1: we train and evaluate our machine learning models using the complete CLIMB data with a nested cross-validation approach. We analyze the experimental results and draw our conclusions, including the efficacy of our models.

Step 2: we conduct the same experiment on the CLIMB data, but only with the set of variables overlapping with the EPIC data. The results will be (1) compared to the ones from step 1 to confirm the robustness of our models, and (2) validated using the EPIC dataset.

Step 3: we conduct our risk factor analysis by extracting the top ten predictive variables from the linear (i.e., SVM and Logistic Regression) and tree-based (i.e., Random Forest, XGboost, and LightGBM) models. Principle risk factors are identified as the common predictors in these algorithms. The same analysis is conducted independently for the two datasets using their complete data. In addition to validating the common risk factors, our study further helps to identify potential key biomarkers that the two datasets failed to collect.

### Baseline models

**Fig. 1:**
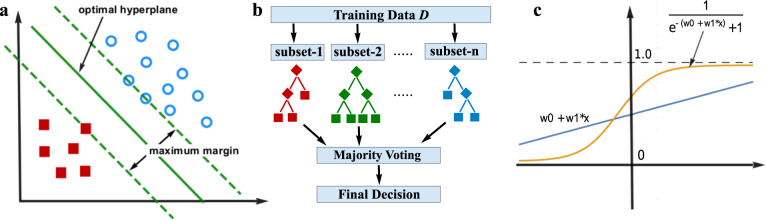
Illustration of three baseline machine learning models. **a** Support Vector Machine: red squares and blue circles represent data from different classes. The optimal decision plane achieves the largest separation, or margin, between the two classes. **b** A Random Forest with *n* decision trees. Each tree is trained with a randomly sampled subset of training data. Predictions from all trees are combined using majority voting to produce a final decision. **c** Logistic Regression with one dependent variable. The blue line is the linear regression model of the observed data. The sigmoid function transforms the linear model’s predictions into values between 0 and 1, which indicate the observations’ likelihood of belonging to the positive class.

We selected the following three established and popular machine learning algorithms as our baseline learners. These methods are illustrated in Fig. 1.

A SVM^18^ performs classification tasks by constructing a decision boundary (i.e., hyperplanes) in a multidimensional space that separates instances of different class labels. As illustrated in Fig. 1a, SVM strives to find a hyperplane that has the maximum margin, i.e., the maximum distance between the hyperplane and the data points of both classes. Maximizing the margin distance reinforces that future data points can be classified with more confidence. SVM is capable of transforming the data into a higher dimensional space, using various kernel functions to enhance data separability. In our study, we adhered to the linear SVM to facilitate risk factor analysis.

A Random Forest^19^ is a collection of DTs. A DT model uses a tree structure to model the data, in which each leaf node corresponds to a class label and attributes are represented as the internal nodes of the tree. Each branch represents a potential value of its parent node (i.e., an attribute). The major challenge in building a DT model is choosing the attribute for each node at each level. Information Gain and Gini Index are the two popular metrics used for attribute selection. DTs tend to have high variance since they are likely to overfit the training data. A Random Forest model, illustrated in Fig. 1b, creates a forest of DTs where each DT is trained with a subset of training instances and a subset of attributes. By pooling predictions from multiple DTs, a Random Forest reduces the variance of each individual DT and achieves a more robust and superior performance. In our study, we used a random forest of 50 DTs, where each tree was built with ten randomly selected attributes. The rest of the model parameters were assigned the default values in Python’s scikit-learn package.

Logistic regression^20^ is a generalized linear model that studies the association between a categorical response variable *Y* and a set of independent (explanatory) variables *X* = {*X*1, *X*_2_, …, *X*_*n*_}. As illustrated in Fig. 1c, the *Y* variable is first modeled as a linear function of *X* with coefficients *W* = {*W*_0_, *W*_2_, …, *W*_*n*_}, and then the predictions (*y*_*i*_’s) are transformed into probability scores using a sigmoid function $f\left( y \right) = \frac{1}{{1 + e^{ - y}}}$. In a binary classification task, the scores indicate a corresponding instance’s likelihood of belonging to the positive class. Thus, a cutoff (e.g., 0.5) can be established as a decision boundary to further categorize the instances into the more likely class. The “training” process involves adjusting the coefficients to maximize the cross-entropy of the model outputs and the true class labels.

### Ensemble models

In addition to individual machine learning algorithms, we explored ensemble techniques^21^ to integrate information from the three base classifiers described above. Ensemble learning is a family of algorithms that seek to create a “strong” classifier based on a group of “weak” classifiers. In this context, “strong” and “weak” refer to how accurately the classifiers can predict the target variable. Ensemble learning has been proven to produce improved and more robust performance than single models.

**Fig. 2:**
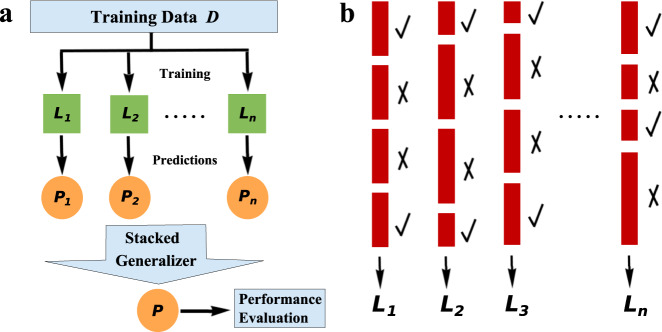
Illustration of ensemble learning and adaptive boosting. **a** Ensemble learning: *L*_*1*_*, L*_*2*_*, …, L*_*n*_ are independent learners trained on the entire training data *D*. The stacked generalizer is a logistic regression model trained to produce a final prediction *P* based on the decisions from individual classifiers. Model performance is measured using the final predictions. **b** Adaptive boosting: checkmarks and crosses indicate correctly and incorrectly classified instances, respectively. The heights of the rectangles are proportional to the weights of the training instances. A sequence of learners, *L*_*1*_*, L*_*2*_*, …, L*_*n*_, is generated with each new model trained on a re-weighted dataset, which boosts the weights of the misclassified training instances in the previous model.

Figure 2a illustrates the principle of ensemble learning. Specifically, multiple base classifiers, *L*_1_, *L*_2_, …, *L*_*n*_, are built for the original classification task with the training data *D*. A Meta-learner *L* is constructed by combining the predictions, *P*_1_, *P*_2_, …, *P*_*n*_, from the base classifiers to improve predictive accuracy. Our Meta-learner *L* is an example of a heterogeneous ensemble because its base learners are obtained from different machine learning algorithms. Our next model, XGBoost^22^, explores the efficacy of a homogeneous ensemble, where the base classifiers are obtained using a single machine learning algorithm. For the task of combining the outcomes from the base learners, we applied stacked generalization^23^, in which an additional linear regression model was trained to predict the target variable in *D* based on the individual predictions from our three baseline classifiers. Stacking typically yields better performance than a straightforward majority voting approach.

We investigated the performance of XGBoost^22^, an algorithm that has gained much popularity and attention since its inception in 2016. XGBoost was the winning algorithm for a number of machine learning competitions. The algorithm belongs to the family of homogeneous ensemble methods, in which the base learners, *L*_1_, *L*_2_, …*, L*_*n*_, are created using a single machine learning algorithm exploiting the concept of “adaptive boosting”^24^. Figure 2b illustrates the concept of “adaptive boosting”. In particular, a sequence of classifiers is generated with the new model aiming to correct the errors made by the previous model. This correction is achieved by boosting the weights of the misclassified training instances in the previous model so that the new model will have a higher likelihood of correctly classifying them. Predictions from these homogenous learners are integrated into a final decision using methods, such as majority voting or stacked generalization^23^. In XGBoost, instead of boosting the weights, the algorithm fits the new model to residuals of the previous model and then minimizes the loss when adding the latest model. The process is equivalent to updating your model with a gradient descent toward a local optimum solution.

The third ensemble learner we employed in our study is LightGBM^25^, a gradient boosting tree-based framework which implements two new techniques: *Gradient-based One-Side Sampling* (GOSS) and *Exclusive Feature Bundling* (EFB). In particular, with GOSS, the algorithm keeps all large gradient instances and only samples from the population of small gradient instances. Thus, GOSS focuses on large gradient instances as they are considered undertrained. With EFB, the algorithm bundles mutually exclusive features (i.e., they rarely take nonzero values simultaneously) to reduce the number of features. Compared to other tree-based algorithms, LightGBM produces much more complex trees by following a leaf-wise split rather than a level-wise split, which is the main factor contributing to LightGBM’s superior performance.

### Reporting summary

Further information on research design is available in the Nature Research Reporting Summary linked to this article.

## Supplementary information

Supplementary Information

Reporting Summary

## Data Availability

Deidentified data will be provided to qualified investigators upon reasonable request.

## Code availability

Models are developed in Python programming language with *pandas*, *sklearn*, and *imblearn* packages. Program code available under GitHub repository https://github.com/tongwangnuliba/Ensemble-Learning-Predicts-Multiple-Sclerosis-Disease-Course.
