# Author Correction: Ensemble learning predicts multiple sclerosis disease course in the SUMMIT study

**DOI:** 10.1038/s41746-020-00361-9

**Published:** 2020-11-20

**Authors:** Yijun Zhao, Tong Wang, Riley Bove, Bruce Cree, Roland Henry, Hrishikesh Lokhande, Mariann Polgar-Turcsanyi, Mark Anderson, Rohit Bakshi, Howard L. Weiner, Tanuja Chitnis, SUMMIT Investigators

**Affiliations:** 1 Department of Computer and Information Science, Fordham University, New York, NY USA; 2 University of California, San Francisco, CA USA; 3 SUMMIT Consortium, Boston, MA USA; 4 SUMMIT Consortium, San Francisco, CA USA; 5 Brigham Multiple Sclerosis Center, Ann Romney Center, Brigham and Women’s Hospital, Harvard Medical School, Boston, MA USA

**Keywords:** Multiple sclerosis, Multiple sclerosis

Correction to: *npj Digital Medicine* 10.1038/s41746-020-00338-8, published online 16 October 2020

The original version of this Article contained an error in the affiliations.

The affiliation University of California, San Francisco, CA, USA was incorrectly listed as University of California, San Francisco, MA, USA.

This has now been corrected in both the PDF and HTML versions of the article.

