# Supplementary material for: Ensemble learning predicts multiple sclerosis disease course in the SUMMIT study
Source: NPJ Digit Med. 2020 Oct 16;3:135. doi: 10.1038/s41746-020-00338-8 (PMC7567781; doi:10.1038/s41746-020-00338-8)
Supplement: Supplementary file 1 — Supplementary Information [file 41746_2020_338_MOESM1_ESM.pdf]

Supplementary Information

SUMMIT Consortium Members

Mark Anderson[3,4], Jalayne Arias[2,4], Rohit Bakshi[3,4], Sergio Baranzini[2,4], Antje Bischof[2,4], Riley Bove[2,4], Tanuja Chitnis[3,4], Bruce Cree[2,4], Bonnie Glanz[3,4], Stephen Hauser[2,4], Brian Healy[3,4], Roland Henry[2,4], Jill Hollenbach[2,4], Robin Lincoln[2,4], Cuquita O'Shea[2,4], Jorge Oksenberg[2,4], Nikolaos Patsopoulos[3,4], Anu Paul[3,4], Mariann Polgar-Turcsanyi[3,4], Francisco Quintana[3,4], Adam Renschen[2,4], Adam Santaniello[2,4], Taylor Saraceno[3,4], Howard Weiner[3,4]
