# Supplementary material for: Ensemble learning predicts multiple sclerosis disease course in the SUMMIT study
Source: NPJ Digit Med. 2020 Oct 16;3:135. doi: 10.1038/s41746-020-00338-8 (PMC7567781; doi:10.1038/s41746-020-00338-8)
Supplement: Supplementary file 2 — Reporting Summary [file 41746_2020_338_MOESM2_ESM.pdf]

nature research

Corresponding author(s): Tanuja Chitnis

Last updated by author(s): Jul 15, 2020

Reporting Summary

Nature Research wishes to improve the reproducibility of the work that we publish. This form provides structure for consistency and transparency in reporting. For further information on Nature Research policies, see our Editorial Policies and the Editorial Policy Checklist.

Statistics

For all statistical analyses, confirm that the following items are present in the figure legend, table legend, main text, or Methods section.

n/a	Confirmed	
☐	☒	The exact sample size (n) for each experimental group/condition, given as a discrete number and unit of measurement
☐	☒	A statement on whether measurements were taken from distinct samples or whether the same sample was measured repeatedly
☒	☐	The statistical test(s) used AND whether they are one- or two-sided<br>*Only common tests should be described solely by name; describe more complex techniques in the Methods section.*
☐	☒	A description of all covariates tested
☒	☐	A description of any assumptions or corrections, such as tests of normality and adjustment for multiple comparisons
☒	☐	A full description of the statistical parameters including central tendency (e.g. means) or other basic estimates (e.g. regression coefficient) AND variation (e.g. standard deviation) or associated estimates of uncertainty (e.g. confidence intervals)
☒	☐	For null hypothesis testing, the test statistic (e.g. F, t, r) with confidence intervals, effect sizes, degrees of freedom and P value noted<br>*Give P values as exact values whenever suitable.*
☒	☐	For Bayesian analysis, information on the choice of priors and Markov chain Monte Carlo settings
☒	☐	For hierarchical and complex designs, identification of the appropriate level for tests and full reporting of outcomes
☒	☐	Estimates of effect sizes (e.g. Cohen's d, Pearson's r), indicating how they were calculated

Our web collection on statistics for biologists contains articles on many of the points above.

Software and code

Policy information about availability of computer code

Data collection	Data for the CLIMB study was collected in a customized Oracle-based database. Data for the EPIC study was collected using standardized electronic clinical research forms.
Data analysis	Models are developed in Python programming language with pandas, sklearn, and imblearn packages. Program code available under GitHub repository https://github.com/tongwangnuliba/Ensemble-Learning-Predicts-Multiple-Sclerosis-Disease-Course

For manuscripts utilizing custom algorithms or software that are central to the research but not yet described in published literature, software must be made available to editors and reviewers. We strongly encourage code deposition in a community repository (e.g. GitHub). See the Nature Research guidelines for submitting code & software for further information.

Data

Policy information about availability of data

All manuscripts must include a data availability statement. This statement should provide the following information, where applicable:

- Accession codes, unique identifiers, or web links for publicly available datasets
- A list of figures that have associated raw data
- A description of any restrictions on data availability

Deidentified data will be provided to qualified investigators upon reasonable request.

Field-specific reporting

Please select the one below that is the best fit for your research. If you are not sure, read the appropriate sections before making your selection.

☒ Life sciences ☐ Behavioural & social sciences ☐ Ecological, evolutionary & environmental sciences

For a reference copy of the document with all sections, see nature.com/documents/nr-reporting-summary-flat.pdf

Life sciences study design

All studies must disclose on these points even when the disclosure is negative.

Sample size	724 patients from the CLIMB dataset; 400 patients from the EPIC dataset.
Data exclusions	All data were included in the analysis.
Replication	Reproducibility of experimental results was confirmed by running the programs five times and achieving stable performance measured by 10-fold cross validation.
Randomization	All experiments were conducted by running a nested cross-validation. Specifically, the outer loop splits the data into 10 stratified non-overlapping folds. Each of the 10 folds will subsequently be held out as the test data while the remaining folds form the training data. For each training set, we apply a nested 5-fold cross-validation to select the hyper-parameters via a grid search based on the highest AUC (Area Under the ROC Curve) score. We report the average model performance of the outer 10 test folds.
Blinding	The investigators were blinded to group allocation during data collection and analysis.

Reporting for specific materials, systems and methods

We require information from authors about some types of materials, experimental systems and methods used in many studies. Here, indicate whether each material, system or method listed is relevant to your study. If you are not sure if a list item applies to your research, read the appropriate section before selecting a response.

Materials & experimental systems

n/a	Involved in the study	
☒	☐	Antibodies
☒	☐	Eukaryotic cell lines
☒	☐	Palaeontology and archaeology
☒	☐	Animals and other organisms
☐	☒	Human research participants
☐	☒	Clinical data
☒	☐	Dual use research of concern

Methods

n/a	Involved in the study	
☒	☐	ChIP-seq
☒	☐	Flow cytometry
☒	☐	MRI-based neuroimaging

Human research participants

Policy information about studies involving human research participants

Population characteristics	The CLIMB dataset has 724 subjects with an average age = 42.83 (SD 10.71); of these, 76.1% are females. The EPIC dataset has 400 subjects with an average age = 42.37 (SD 9.73); of these, 66.8% are females.
Recruitment	Subjects are recruited into the CLIMB and EPIC studies from the MS clinics at these sites. All eligible MS patients are offered participation in studies.
Ethics oversight	IRBs were approved by the Partners Human Research Committee and Fordham University.

Note that full information on the approval of the study protocol must also be provided in the manuscript.

Clinical data

Policy information about clinical studies

All manuscripts should comply with the ICMJE guidelines for publication of clinical research and a completed CONSORT checklist must be included with all submissions.

Clinical trial registration	NA
Study protocol	The full study protocol for CLIMB is located by the Partners Human Research Committee; The

Data collection	Data is collected in the BWH and UCSF clinics by trained study coordinators and clinicians. Data is inputted into standardized CRFs.
Outcomes	Outcomes were defined based on key outcomes defined for multiple sclerosis identified through the current literature
